# Determination of the fit growth curve model and relationships among growth parameters by nonlinear functions in Kaçkar hair goats

**DOI:** 10.5194/aab-68-497-2025

**Published:** 2025-07-28

**Authors:** Sadrettin Yüksel, Fatma Yüksel, Erdoğan Sezgin

**Affiliations:** 1 Department of Animal Science, Faculty of Agriculture, Atatürk University, 25070 Erzurum, Türkiye; 2 Aegean Agricultural Research Institute, 35660 İzmir, Türkiye; 3 Departmant of Animal Science, Eastern Anatolian Agriculture Institute, 25090 Erzurum, Türkiye

## Abstract

The objectives of this study are to identify a suitable mathematical model for determining the breeding strategies and describing the growth curve of Kaçkar hair goat based on data at birth, at 3 months of age, 6 months of age, 9 months of age, and 12 months of age using records of body weight (BW), body length (BL), height at withers (HW), and chest circumference (CC) from birth to 12 months of age. Data of 242 Kaçkar hair goats as part of the National Project on Conservation and Sustainable Utilization of Domestic Animal Genetic Resources, on one farm, were recorded during the years 2018–2021 and analyzed for estimating growth curve parameters. The growth models were compared using the Bayesian information criterion (BIC), Akaike's information criterion (AIC), adjusted coefficient of determination ( $R_{\mathrm{adj}}^{2}$ ), and root mean square error (RMSE) based on the parameters of the growth curve such as the asymptote for the size measured ( A ), rate of gain ( b ), and growth rate – constant of integration ( k ). For the Brody model, we estimated $R_{\mathrm{adj}}^{2}$ for body weight, body length, height at withers, and chest circumference that was 86.0 %, 85.2 %, 84.9 %, and 85.3 % in males and 86.0 %, 78.8 %, 79.7 %, and 84.8 % in females, respectively. For the Von Bertalanffy model, we also estimated $R_{\mathrm{adj}}^{2}$ for same parameters that were 87.6 %, 85.7 %, 87.5 %, and 87.1 % in males and 85.9 %, 78.3 %, 82.6 %, and 80.2 % in females, respectively. Among all used models, the Brody for females and Von Bertalanffy for males resulted in the smallest AIC, BIC, and RMSE values. As a result, growth curve models are valuable instruments for determining the profitable slaughter age, and they provide practical information on the rearing practices of both sexes of Kaçkar hair goats for meat production. Thus, it could be applied to rearing programs for early selection, enabling breeding strategies when needed.

## 1 Introduction

Growth, that is, changes during the life of an animal, from the embryonic stage up to adult age or mature weight (Bahreini Behzadi et al., 2014) could be characterized by the growth rate or by weight and size increases during different physiological periods. Results achieved in this process may change depending on varied components, such as the species and breed of animal and environmental factors (Kheirabadia and Rashidib, 2019). So, knowledge can aid in directly choosing animals to improve growth traits (Sunwasiya, 2022). In this context, in order to explain the course of growth, some mathematical functions that are asymptotic and determined by a maximum size, which is reached with the growth rate diminishing, have been improved. The Brody, Gompertz, Von Bertalanffy, and logistic models are some of these nonlinear models used to estimate growth curve parameters (Arré et al., 2019). As for different species of livestock (Ozturk et al., 2023; Soysal et al., 2015), these nonlinear models were used to determine the growth curve parameters also in Beetal goats (Magotra et al., 2021). In phylogenetic analyses, the Kaçkar hair goat, which is associated with the breeds in the northern regions, is widespread in the northern region line and inland regions of Türkiye (Yüksel et al., 2020).

To the best on our knowledge, there has not been a systematic study on the estimation of growth in the Kaçkar breed using nonlinear growth curve models. Therefore, this study aimed to find the best-fitted nonlinear models to determine a profitable shape of the growth curve from birth to 1 year of age in Kaçkar hair goats.

## 2 Material and methods

### 2.1 Data description

The study was conducted using data on body weight, body length, height at withers, and chest circumference from a total of 242 (141 female and 101 male) Kaçkar hair goat for the National Project on Conservation and Sustainable Utilization of Domestic Animal Genetic Resources conducted by the Eastern Anatolian Agricultural Research Institute. The records were obtained in the Artvin, Hopa, and Hendek villages, northeast region of Türkiye, from 2018 to 2021. The goal in the region was, generally, managed by following the conventional rearing practices. The mating period started between September and October and included at most three estrous cycles (54 d). Births commenced at the beginning of February and ended at the end of March. The kids were fed on natural pastures and kept together with their mothers until the mean weaning age of all kids at about 3 months. The all goats were kept on pastures during the year (indoors for about 15–20 d only in winter by feeding dry hay). All animals were measured and weighted with 0.1 kg accuracy at birth and 3 (weaning), 6, 9, and 12 months of age, and the data were recorded. However, 9- and 12-month body weights were estimated by measuring the chest circumference with the help of a measuring tape (Chacón-Hernández and Boschını-Figueroa, 2017) as the animals were in high plateaus.

### 2.2 Statistical analysis

Four nonlinear models that are in the following were used for fitting growth curve characteristics for males and females separately.

The first model is the Brody model (Brody, 1945):

1 $$Y(t)=A(1-be^{-kt})+\varepsilon .$$

The following model is the Gompertz model (Laird, 1965):

2 $$Y\left(t\right)=Ae^{-b(\mathrm{exp}\left(-kt\right))}+\varepsilon .$$

The third model is the logistic model (Nelder, 1961):

3 $$Y\left(t\right)=\frac{A}{1+be^{\left(-kt\right)}}+\varepsilon .$$

The final model is the Von Bertalanffy model (Von Bertalanffy, 1957):

4 $$Y\left(t\right)=A(1-be^{\left(-kt\right)}{)}^{3}+\varepsilon ,$$

where Y(t) is the size measured at age t in days, A is the asymptote for the size measured – mature live weight (body weight, body length, height at the withers, and chest circumference), t is the age of animal at the time of scaling, b is the ratio of gained weight after birth to mature weight (kg or cm), k is the growth rate from birth to maturity, and ε is the fitting error. The estimation of parameters with the standard error for these models was undertaken using the NLIN procedure in SPSS.25.0 (SPSS, 2021).

The nonlinear models for fit statistics were determined with the Bayesian information criterion (BIC), Akaike's information criterion (AIC), adjusted coefficient of determination ( $R_{\mathrm{adj}}^{2}$ ), and root mean square error (RMSE).

5BIC=nlnRSSn+pln(n),6AIC=nln(RSS)+2p,7RMSE=RSSn-p-1,

where n , RSS, and p are the number of observations (data points), the residual sum of squares, and the number of parameters in the equation, respectively. Smaller values of BIC, AIC, and RMSE suggest a better fit when comparing the models.

The adjusted coefficient of determination ( $R_{\mathrm{adj}}^{2}$ ) is as follows:

8 $$R_{\mathrm{adj}}^{2}=1-\frac{\text{RSS}/\text{dfe}}{\text{TSS}/\text{dft}},$$

where TSS is the total sum of squares, dft is the degrees of freedom ( n-1 ) of the estimate of the population variance of the dependent variable, and dfe is the degrees of freedom ( n-p-1 ) of the estimate of the underlying population error variance. The model sum of squares plus the error sum of squares equals the total sum of squares. A higher value of the adjusted coefficient of determination indicates the best-fit model.

On the other hand, when studying genetic parameters of growth curve characteristics, a univariate animal model given in the following was employed which considered the fixed effects of the sex of the goats (male and female) and random effects of animal according to the average information-restricted maximum likelihood method (AI-REML) using Wombat software (Meyer, 2006):

9 Y=Xβ+Zu+e,

where Y is the observed value vector of all traits; X is the structure matrix of fixed effects; β is the fixed-effect vector, including sex; Z is the structure matrix of random effects; u is the individual additive-effect vector; and e is the random residual-effect vector.

The estimation of genetic and phenotypic correlations was done using a bivariate animal model.

## 3 Results

Estimated growth curve parameters for the Kaçkar goat breed by some nonlinear growth models are presented in Tables 1 and 2 for males and females, respectively. In the tables are the estimated growth parameters ( A , b , and k ) obtained by the Gompertz, logistic, Brody, and Von Bertalanffy models, as well as the coefficient of determination ( $R^{2}$ ), for some physical characteristics, such as body weight, height at withers, body length, and chest circumference (Table 1).

In males, the coefficient of determination ( $R^{2}$ ), which determines of degree of confidence of the body weight was observed for the Gompertz, logistic, Brody, and Von Bertalanffy models to be 87.4 %, 87.3 %, 92.6 %, and 92.8 %, respectively, and had the highest-value for the Von Bertalanffy model. Similarly, $R^{2}$ values that were estimated by Von Bertalanffy model for BL, HW, and CC characteristics were higher and were 91.3 %, 89.6 %, and 93.7 %, respectively.

In females, estimated asymptotic growth curve parameters of parameters A , b , and k and the coefficient of determination of the nonlinear mathematical models are presented in Table 2. The highest $R^{2}$ (96.7 %) value for asymptote values of BW characteristic was in the Brody model, and the value was higher than those of the Gompertz, logistic, and Von Bertalanffy models at 96.0 %, 96.0 %, and 96.1 %, respectively. Similar results were also observed for BL, HW, and CC characteristics for the Brody model. $R^{2}$ values for BL, HW, and CC characteristics estimated by the Brody model were higher than those of other models and were 94.6 %, 96.2 %, and 94.8 %, respectively.

**Table 1:** Estimated growth curve parameters for male Kaçkar goats from different nonlinear models.

Model	Trait	Parameter			
		A	b	k	$R^{2}$
Gompertz	BW (kg)	47.03±2.22	3.02±0.01	0.63±0.10	87.4
	BL (cm)	69.18±3.43	2.91±0.02	0.14±0.01	87.8
	HW (cm)	68.26±2.11	1.92±0.01	0.43±0.01	88.1
	CC (cm)	82.43±3.38	2.27±0.03	1.09±0.01	87.4
Logistic	BW (kg)	46.42±3.13	3.30±0.01	0.16±0.21	87.3
	BL (cm)	67.06±2.14	2.59±0.01	0.60±0.01	85.9
	HW (cm)	68.81±3.33	2.37±0.02	0.22±0.02	85.7
	CC (cm)	81.40±1.92	2.68±0.02	0.14±0.11	86.1
Brody	BW (kg)	54.81±3.05	1.29±0.01	0.32±0.01	92.6
	BL (cm)	73.14±3.21	1.32±0.01	0.17±0.01	90.6
	HW (cm)	76.32±4.90	0.89±0.02	0.08±0.01	88.1
	CC (cm)	85.82±3.08	0.94±0.38	0.09±0.02	92.3
Von Bertalanffy	BW (kg)	52.30±2.71	1.45±0.01	0.10±0.02	92.8
	BL (cm)	70.33±1.96	0.91±0.01	0.13±0.02	91.3
	HW (cm)	74.01±1.63	1.29±0.02	0.16±0.01	89.6
	CC (cm)	83.28±2.31	0.99±0.11	0.07±0.01	93.7

BW: body weight, BL: body length, HW: height at withers, CC: chest circumference, A : asymptote for the size measured – mature live weight, b : rate of gained weight after birth to mature weight (kg or cm), k : growth rate from birth to maturity.

The results of model comparison for the growth curve of Kaçkar male and female goats tested according to the Gompertz, logistic, Brody, and Von Bertalanffy models based on the goodness-of-fit measures of AIC, BIC, RMSE, and $R_{\mathrm{adj}}^{2}$ are indicated in Table 3. Among all compared models, the smallest AIC and BIC values were, mostly, determined by the Von Bertalanffy for males and Brody for females. The AIC value determined for HW was lower in the Gompertz model than other models for females. The logistic model that was lower than Gompertz for BW and BL in females was, mostly, the improper model with the highest AIC and BIC values. The Von Bertalanffy model provided the lowest RMSE values for males among used models, but this model had a higher RMSE value compared to Brody models for female goats. The high $R_{\mathrm{adj}}^{2}$ values determined for the characters evaluated according to the models discussed were not concentrated on a single model. It was determined that they are distributed among the Gompertz (BL for males and BW and CC for females) and Von Bertalanffy models (all other characteristics).

**Table 2:** Estimated growth curve parameters for female Kaçkar goats from different nonlinear models.

Model	Trait	Parameter			
		A	b	k	$R^{2}$
Gompertz	BW (kg)	36.99±2.62	1.21±0.01	0.11±0.01	96.0
	BL (cm)	62.21±287	1.86±0.12	0.14±0.01	94.1
	HW (cm)	65.86±2.72	1.86±0.20	0.05±0.02	89.8
	CC (cm)	76.76±2.21	1.76±0.02	0.28±0.01	93.1
Logistic	BW (kg)	36.81±1.91	2.83±0.22	0.62±0.01	96.0
	BL (cm)	62.11±2.26	3.08±0.33	0.22±0.20	91.1
	HW (cm)	63.01±2.90	2.33±0.61	0.12±0.03	93.2
	CC (cm)	75.46±3.00	2.58±0.80	0.51±0.01	92.8
Brody	BW (kg)	42.27±2.33	1.19±0.32	0.52±0.02	96.7
	BL (cm)	67.22±0.01	3.00±0.02	1.03±0.01	94.6
	HW (cm)	66.18±2.12	2.01±0.23	0.33±0.20	96.2
	CC (cm)	79.44±2.92	2.17±0.37	0.21±0.01	94.8
Von Bertalanffy	BW (kg)	39.00±2.26	0.62±0.01	0.19±0.02	96.1
	BL (cm)	64.24±2.19	2.14±0.29	0.12±0.01	92.0
	HW (cm)	62.80±2.27	1.27±0.10	0.06±0.01	94.4
	CC (cm)	78.58±2.55	1.55±0.20	0.11±0.01	93.7

BW: body weight, BL: body length, HW: height at withers, CC: chest circumference, A : asymptote for the size measured – mature live weight, b : rate of gained weight after birth to mature weight (kg or cm), k : growth rate from birth to maturity.

**Table 3:** Model ranking and model goodness of fit estimators of nonlinear functions to describe the growth curve of Kaçkar goats.

Model	Trait	Fit statistics (male/female)			
		AIC	BIC	RMSE	$R_{\mathrm{adj}}^{2}$
Gompertz	BW (kg)	16.421/18.312	12.120/14.022	3.17/2.54	85.6/87.6
	BL (cm)	13.728/15.871	11.089/11.819	4.21/3.99	87.2/83.8
	HW (cm)	13.819/15.183	9.942/11.131	4.35/4.03	84.6/78.1
	CC (cm)	17.943/19.809	13.343/14.184	4.61/4.27	82.9/85.7
Logistic	BW (kg)	16.902/18.052	11.202/14.511	3.84/2.47	84.2/87.2
	BL (cm)	14.002/15.671	10.222/11.578	4.60/4.08	82.5/84.0
	HW (cm)	13.896/15.904	9.136/11.484	4.71/4.13	87.5/77.6
	CC (cm)	18.527/20.194	14.257/16.941	4.70/4.28	85.5/86.7
Brody	BW (kg)	16.388/17.717	11.128/13.317	3.66/2.10	86.0/86.0
	BL (cm)	13.419/14.989	11.909/10.719	3.74/2.73	85.2/78.8
	HW (cm)	13.271/15.291	9.649/10.293	3.53/2.30	84.9/79.7
	CC (cm)	17.381/19.873	13.891/14.393	3.91/2.28	85.3/84.0
Von Bertalanffy	BW (kg)	16.201/18.001	11.120/15.014	3.16/2.45	87.6/85.9
	BL (cm)	13.627/15.001	8.282/12.998	2.92/3.19	85.7/78.3
	HW (cm)	13.096/15.649	8.621/12.453	2.88/3.24	87.5/82.6
	CC (cm)	17.713/19.391	12.139/15.187	2.41/3.76	87.1/80.2

BW: body weight, BL: body length, HW: height at withers, CC: chest circumference.

**Figure 1:**
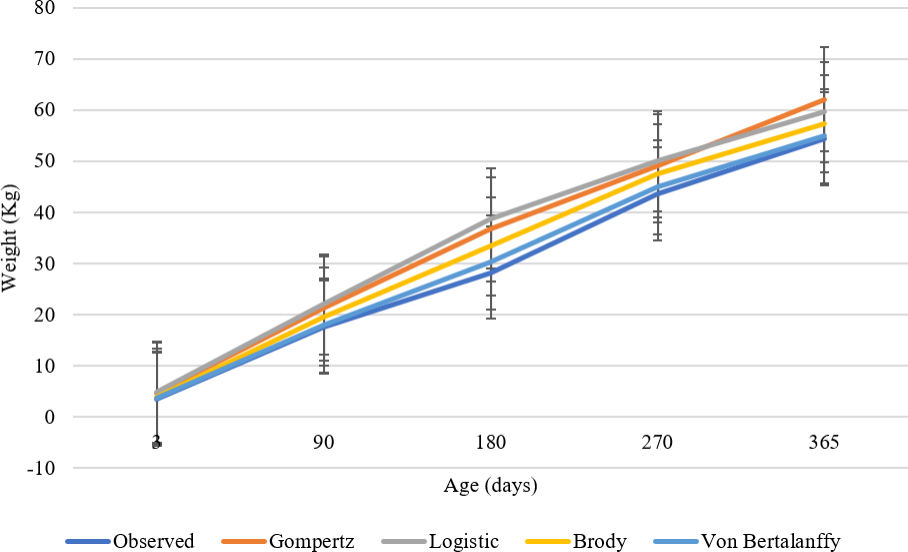
Actual and predicted body weights as a function of age obtained with models used for male Kaçkar goats.

**Figure 2:**
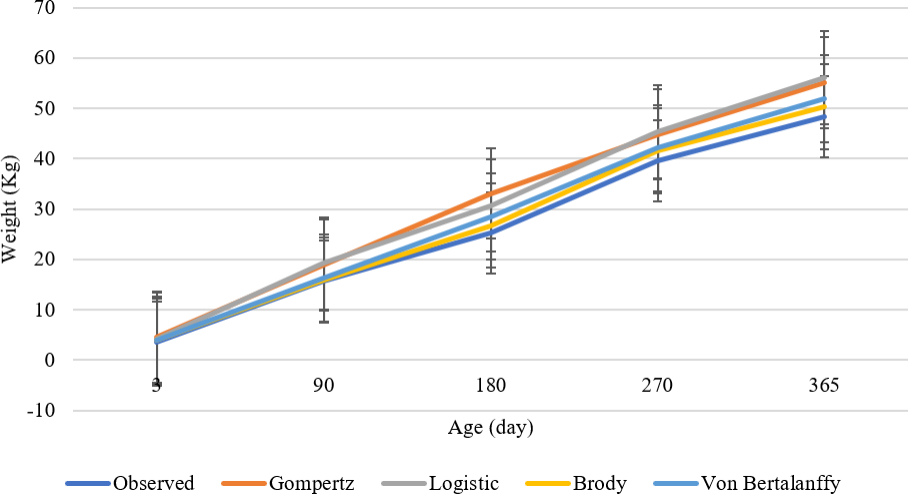
Actual and predicted body weights as a function of age obtained with models used for female Kaçkar goats.

Graphics of growth curves and their prediction limits of the evaluated traits based on the Gompertz, logistic, Brody, and Von Bertalanffy models are represented for males and females in Figs. 1 and 2, respectively.

The estimates of variance components, additive heritability of studied growth curve traits and estimates of genetic and phenotypic correlations among growth curve in Kaçkar hair goat were indicated in Tables 4 and 5.

**Table 4:** Estimates of variance components and of additive heritability ratio of growth curve traits in Kaçkar hair goat.

Variance component	Trait	A	b	k
Additive variance	BW	29.21	0.00064	0.00033
	CC	41.82	0.00047	0.00009
Residual variance	BW	57.18	0.00058	0.00290
	CC	89.06	0.00042	0.00094
Phenotypic variance	BW	86.39	0.00122	0.00323
	CC	130.88	0.00089	0.00103
Additive heritability	BW	0.46±0.002	0.41±0.002	0.38±0.001
	CC	0.37±0.001	0.39±0.001	0.36±0.002

BW: body weight, CC: chest circumference, A : asymptote for the size measured – mature live weight, b : rate of gained weight after birth to mature weight (kg or cm), k : growth rate from birth to maturity.

The phenotypic variance for A , B , and k according to BW was observed to be 63.09, 0.00322, and 0.00355, respectively, and for the same parameter according to CC, it was also 96.11, 0.00235, and 0.00075. The estimates of additive heritability of A , b , and k parameters for BW were observed to be 0.46±0.002 , 0.41±0.002 , and 0.38±0.001 , respectively, while according to CC, they were 0.37±0.001 , 0.39±0.001 , and 0.36±0.002 .

**Table 5:** Estimates of genetic and phenotypic correlations among growth curve traits in Kaçkar hair goat.

Parameter	Trait	Genetic correlation ( $r_{g}$ )	Phenotypic correlation ( $r_{p}$ )
A-b	BW	0.97±0.02	0.71±0.03
	CC	0.96±0.04	0.66±0.07
A-k	BW	-0.93±0.07	-0.65±0.03
	CC	-0.97±0.11	0.69±0.07
b-k	BW	0.91±0.28	-0.52±0.05
	CC	-0.91±0.03	-0.47±0.01

BW: body weight, CC: chest circumference, A : asymptote for the size measured – mature live weight, b : rate of gained weight after birth to mature weight (kg or cm), k : growth rate from birth to maturity.

The estimates of genetic correlations among A-b , A-k , and b-k for BW were 0.97±0.02 , -0.93±0.07 , and 0.91±0.28 , respectively, while for CC, they were 0.96±0.04 , -0.97±0.11 , and -0.91±0.03 . The estimates of phenotypic correlations among A-b , A-k , and b-k for BW were 0.71±0.03 , -0.65±0.03 , and -0.52±0.05 , respectively, and for CC, they were 0.66±0.07 , 0.69±0.07 , and -0.47±0.01 .

## 4 Discussion

Lately, growth curve studies have been intensively used in order to make improvement and to identify strategies in the profitable use of livestock (Hossein-Zadeh, 2015) based on some models. Growth is a characteristic defined as an increase in weight or body measurements and can be measured directly on the animal defined. For this process, some mathematical models have been used to comment on the biological potential of animals at variable scales (Waheed et al., 2011). It is inevitable to have to determine the compatibility of these models in rightly, as the goodness of fit is an important criteria used to compare the models.

In the current study, the Von Bertalanffy model displays the best fit for evaluations of males, determining the highest association between estimated and observed weights, and it was asserted that it could be used to explain the growth process and development of the studied male Kaçkar goat breed. As for females, the Brody model stood out as the most appropriate model in terms of considered criterions. On the other hand, following the logistic model, the Gompertz model, in terms of some characteristics, indicated the most unproductive fit of growth in male and female Kaçkar goats due to the highest AIC, BIC, and RMSE values and the lowest $R_{\mathrm{adj}}^{2}$ values. A study on Beetal and south African breeds showed that the Von Bertalanffy model effectively explained the $R_{\mathrm{adj}}^{2}$ value for the growth curve of Beetal goats (Hossein-Zadeh, 2025). Magotra et al. (2021) evaluated the growth curve of Beetal goats in India by birth, weaning (3-month), 6-month, 9-month, and 12-month body weight using five models, i.e., Brody, Bertalanffy, logistic, Gompertz, and negative exponential, and suggested the Brody model as the best fit for explaining the growth in their resource population. This declaration supported our findings that was determined for females in our study. It was used Gompertz, logistic, Brody, Von Bertalanffy, monomolecular, negative exponential, and Richard models by Tesema et al. (2023). The Brody method was declared, which coincided with our results for females, the best fit in the first filial generation, second filial generation, female third filial generation, and male Boer × Central Highland goats. In the same context, the Brody followed by the Von Bertalanffy model provided the lowest AIC, BIC, and RMSE values, and these functions had the highest $R_{\mathrm{adj}}^{2}$ value compared to other models for F_1_, F_2_, and male goats (Tesema et al., 2023), which supports the findings for male and female of our study. The same researchers declared that the models that best described the style of the growth in F_3_ and female Boer × Central Highland goats were the Brody and Richard models. On the other hand, the Brody model provided the greatest $R^{2}$ values for male and female Sirohi when compared with the Gompertz, logistic, Richard, and Weibull models (Waiz et al., 2019), and the results were in accordance with our findings for females. The Brody and Gompertz models provided the best fit for growth curve estimates in Beetal goats (Waheed et al., 2011). In line with the current finding, Freitas (2005) reported that the Brody and Von Bertalanffy models were more versatile when fitting the growth curve in sheep. The Brody and Von Bertalanffy models were found to be more suitable for growth curves in male Kivircik sheep under a semi-intensive production system (Ozturk et al., 2023). Hossein-Zadeh (2015) evaluated Brody, logistic, Richard, negative exponential, Von Bertalanffy, and Gompertz models and found that the Richard model best described growth in male and female Shall sheep. On the other hand, Tyasi et al. (2022) reported that the Gompertz 3P model was the best model to describe the growth curve of south African nondescript both male and female indigenous goats among using models such as Gompertz 3P, Gompertz 4P, logistic 3P, logistic 4P, and logistic 5P.

Although is an important source for determining the goodness of fit and shape of growth curves, there is relatively little literature information on the nonlinear growth models based on body measurements in goats. In this study, it is determined that the best-fit model was the Von Bertalanffy for males and Brody for females, respectively, regarding the AIC, BIC, and RMSE parameters obtained from Gompertz, logistic, Brody and Von Bertalanffy models and some physical traits, such as the body length, body height, and chest circumference. In a previous study, the determination coefficients of logistic and Gompertz models were the same for the body length of Katjang × Boer does (Hifzan et al., 2024). When our study was evaluated according to these two models, it was seen that the $R_{\mathrm{adj}}^{2}$ value for HW and CC characteristics in the logistic model was higher for males. On the other hand, Kor et al. (2006) reported that the Gompertz model had the highest RMS and the lowest $R_{\mathrm{adj}}^{2}$ value model when comparing various growth models in female Akkeci (white goat). This result did not coincide with our findings based on the Gompertz model as it was lower than the Von Bertalanffy, Brody, and logistic models in our study. Ozturk et al. (2023) found that the logistic, Gompertz, Brody, and Von Bertalanffy models were approximately 0.72 the $R_{\mathrm{adj}}^{2}$ value in Kivircik sheep under a semi-intensive production system. This ratio was lower than our results using the same models.

In the present study, the $R_{\mathrm{adj}}^{2}$ values of HW in the logistic and Von Bertalanffy models are the same for males and higher than those in the Gompertz and Brody models. On the other hand, the $R_{\mathrm{adj}}^{2}$ value estimated by logistic model for females was the highest, and that estimated by the logistic model was the lowest. The Von Bertalanffy and Brody models were also found to be advantageous for AIC, BIC, and RMSE values of HW and were viewed as being the best-fit models. Kor et al. (2006) reported that Gompertz was the best model that was among the models used by us as well, comparing various growth models based on HW in female Akkeci (white goat). Similarly, Hifzan et al. (2024) reported an $R^{2}$ value that was estimated by the Gompertz model for HW as having higher value while comparing Gompertz and logistics growth models in female Katjang × Boer goats in Malaysia. The Gompertz model, which was found to be a suitable model in both studies, followed Von Bertalanffy, Brody, and logistic models, respectively, in our study. On the other hand, based on the AIC value, for females, Von Bertalanffy followed by Brody were determined to be models with the best fit for the CC characteristic. However, the lowest BIC and RMSE and the highest $R_{\mathrm{adj}}^{2}$ values were observed for the same characteristic in the Von Bertalanffy model. The fitting state of the Gompertz model observed based on CC in the present study indicated similar results to a study carried out using different growth models for Akkeci (white goat) (Kor et al., 2006); thus, $R^{2}$ values were low and AIC and BIC values high in both studies. In another study, Hifzan et al. (2024) reported higher $R^{2}$ value for logistic while comparing Gompertz and logistics models in female Katjang × Boer goats. While using the Brody and Von Bertalanffy models, it should be considered that the models tend to sensitively determine the some period weights and the final weights. However, these models, or any other models, cannot be expected to describe all the actual growth curves since animals may not show their potential growth due to environmental effects (Ozturk et al., 2023). Despite everything, growth curves could be accepted as a fit instrument for following animal growth and defining the size of potential deviations from the expected growth performance.

The estimation of variance components and the direct heritability of growth curve parameters based on BW and CC traits in Kaçkar hair goats revealed that heritability could have a normal posterior distribution. The middle heritability of the mature BW and CC might be attributed to there being no dominant effects of environmental factors on Kaçkar hair goats. Thus, some factors such as management and environmental conditions could affect the estimated hereditary values (Li et al., 2024). The direct genetic correlation coefficient between A and k in the body weight growth curve was high, revealing a high negative correlation, which indicated that the growth and development rates were high. Li et al. (2024) reported that the smaller the K , the higher the mature weight of the animal, and in practice, the maturity weight can be increased by reducing the growth rate. On the other hand, the direct genetic correlation coefficient between A-b and b-k was high and positive and negative value, respectively. This result was an identifier of the fact that the growth rate was very high (Li et al., 2024). Also, in a study conducted on Murciano-Granadina goats, positive and medium estimates were obtained for phenotypic and genetic correlations between parameters A and b (Mokhtari et al., 2023). Iqbal et al. (2021) reported, contrary to our findings, a negative genetic correlation between A-k and a positive correlation between b-k . Lambe et al. (2006) reported that A-k was to have medium positive genetic correlations with body weight at 6 and 12 months of age. This finding was not consistent with that of the present study. A similar result was reported for b-k in CC. Contrary to our findings, Li et al. (2024) reported a positive low correlation value.

## 5 Conclusion

The Von Bertalanffy model for males and the Brody model for females were more multifaceted to fit the growth curve in Kaçkar hair goats. The result based on the best-fit growth model indicated that observed values with estimated values for BW, BL, HW, and CC were close. So, the Von Bertalanffy model tends to agree with the Brody model. Females and males had similar realistic values the weight, although there was a reaction against the best-fit model in terms of estimation among male and female goats. The growth curve can save time and cost, enabling the selection, breeding, and marketing of goats based on early maturation parameters. Therefore, the Von Bertalanffy and Brody models can be used to predict the mature body weight and maturation rate of Kaçkar hair goats using body weight and some body measurements and help to determine management and breeding strategies.

## Data Availability

The datasets generated are available from the corresponding author on request.
